# Clubroot Symptoms and Resting Spore Production in a Doubled Haploid Population of Oilseed Rape (*Brassica napus*) Are Controlled by Four Main QTLs

**DOI:** 10.3389/fpls.2020.604527

**Published:** 2020-12-17

**Authors:** Andrea Botero-Ramírez, Anne Laperche, Solenn Guichard, Mélanie Jubault, Antoine Gravot, Stephen E. Strelkov, Maria J. Manzanares-Dauleux

**Affiliations:** ^1^Department of Agricultural, Food and Nutritional Sciences, Faculty of Agricultural, Life and Environmental Sciences, University of Alberta, Edmonton, AB, Canada; ^2^Institut de Génétique, Environnement et Protection des Plantes, INRAE, Institut Agro, Université de Rennes 1, Le Rheu, France

**Keywords:** oilseed rape, *Plasmodiophora brassicae*, clubroot, linkage analysis, quantitative resistance, *Rhizaria*

## Abstract

Clubroot, caused by *Plasmodiophora brassicae* Woronin, is one of the most important diseases of oilseed rape (*Brassica napus* L.). The rapid erosion of monogenic resistance in clubroot-resistant (CR) varieties underscores the need to diversify resistance sources controlling disease severity and traits related to pathogen fitness, such as resting spore production. The genetic control of disease index (DI) and resting spores per plant (RSP) was evaluated in a doubled haploid (DH) population consisting of 114 winter oilseed rape lines, obtained from the cross ‘Aviso’ × ‘Montego,’ inoculated with *P. brassicae* isolate “eH.” Linkage analysis allowed the identification of three quantitative trait loci (QTLs) controlling DI (PbBn_di_A02, PbBn_di_A04, and PbBn_di_C03). A significant decrease in DI was observed when combining effects of the three resistance alleles at these QTLs. Only one QTL, PbBn_rsp_C03, was found to control RSP, reducing resting spore production by 40%. PbBn_rsp_C03 partially overlapped with PbBn_di_C03 in a nucleotide-binding leucine-rich repeat (NLR) gene-containing region. Consideration of both DI and RSP in breeding for clubroot resistance is recommended for the long-term management of this disease.

## Introduction

Clubroot, caused by the obligate parasite *Plasmodiophora brassicae* Woronin, is one of the most important diseases of cruciferous crops worldwide, causing significant yield and quality losses in oilseed rape (canola; *Brassica napus* L.) and other Brassicas (Dixon, 2009). A soilborne disease, clubroot is associated with the formation of large galls on the roots of susceptible hosts, which impede water and nutrient uptake. The life cycle of *P. brassicae* consists of three main stages: (i) survival in the soil, (ii) root hair infection, and (iii) cortical infection. The pathogen survives in the soil as long-lived resting spores (Kageyama and Asano, 2009). Under favorable conditions, the resting spores germinate to produce primary zoospores, which infect the host root hairs. The presence of plant root exudates can enhance resting spore germination (Macfarlane, 1970; Friberg et al., 2005; Rashid et al., 2013). Primary plasmodia develop within the infected root hairs, eventually giving rise to secondary zoospores. The secondary zoospores penetrate the cortical tissue and develop into intracellular secondary plasmodia, which cleave to produce a new generation of resting spores (Kageyama and Asano, 2009). As the galls decompose, these resting spores are released back into the soil, where they serve as inoculum for future infections. As many as 1 × 10^10^ resting spores per g of gall tissue may be produced on a susceptible *B. napus* host (Hwang et al., 2013).

The persistence of *P. brassicae* resting spores in the soil (Wallenhammar, 1996) makes clubroot a particularly difficult disease to manage. Various strategies have been proposed, including crop rotation, biological control, liming of the soil, and manipulation of the seeding date (Hwang et al., 2014). The most effective strategy for clubroot management, however, is the deployment of CR host cultivars (Rahman et al., 2014). The resistance in most CR oilseed rape cultivars is monogenic and derived from the European winter oilseed rape ‘Mendel’ (Diederichsen et al., 2014; Fredua-Agyeman et al., 2018). ‘Mendel’ was one of the first CR winter oilseed rape cultivars with acceptable agronomic performance released in Europe (Diederichsen et al., 2003, 2009). The clubroot resistance in ‘Mendel’ is based on one dominant and race- or pathotype-specific gene (Diederichsen et al., 2006). While genetic resistance is the most effective and convenient method to manage clubroot, the selection pressure it imposes on *P. brassicae* populations can cause rapid shifts in virulence. These shifts may result in a loss of resistance, as has already been documented in oilseed rape/canola in Europe (Diederichsen et al., 2014; Orgeur et al., 2016) and Canada (Strelkov et al., 2016, 2018).

The diversification and introduction of novel resistance sources can reduce the risk of resistance loss, as can the development of cultivars with polygenic resistance and the implementation of strategies such as gene pyramiding and the planting of multilines or cultivar mixtures (Parlevliet and Zadoks, 1977; Pink and Puddephat, 1999). For example, a combination of major resistance genes and quantitative trait loci (QTLs) could result in CR cultivars with resistance that is more durable. The development of cultivars with more diverse resistance, however, requires a deeper understanding of the genetic control of clubroot resistance (Manzanares-Dauleux et al., 2000).

Most genetic studies of clubroot resistance have focused on understanding the resistance harbored in *Brassica oleracea* and *Brassica rapa*, the ancestral parents of *B. napus.* In *B. rapa*, multiple major genes controlling clubroot resistance have been identified, including *Crr1a, Crr1b* (*Crr1a* and *Crr1b* were initially identified as a single locus, *Crr1*), *Crr2*, *Crr3*, *Crr5A*, *CRa*, *CRb*, *CRd*, *CRs*, *Rcr1*, *Rcr2*, *Rcr3*, *Rcr5*, and *Rcr9*^WA^ (Matsumoto et al., 1998; Suwabe et al., 2003; Hirai et al., 2004; Saito et al., 2006; Hatakeyama et al., 2013, 2017; Chu et al., 2014; Huang et al., 2017, 2019; Yu et al., 2017; Nguyen et al., 2018; Pang et al., 2018; Laila et al., 2019; Karim et al., 2020). In addition, two QTLs were reported to control resistance to a Korean isolate of *P. brassicae* classified as per Williams (1966) pathotype 2 (Choi et al., 2020), and two QTLs were found to control resistance to a Chinese isolate of pathotype 7 (Zhu et al., 2019). Another QTL (*Rcr4*) was found to control resistance to Canadian isolates of the pathogen representing pathotypes 2, 3, 5, 6, and 8, classified as per Williams (1966), and two QTLs (*Rcr8* and *Rcr9*) controlled resistance to an isolate of pathotype 5X, as defined on the Canadian Clubroot Differential set (Yu et al., 2017; Strelkov et al., 2018). Three loci (*Crr4*, *CRc*, and *CRk*) controlled resistance to non-pathotyped isolates of *P. brassicae* (Suwabe et al., 2006; Sakamoto et al., 2008). In *B. oleracea*, clubroot resistance has been found to be quantitative and is controlled mainly by QTLs with both major and minor effects (Rocherieux et al., 2004; Neik et al., 2017).

Genetic analyses of the control of clubroot resistance in *B. napus* have identified the presence of a major resistance gene (*Cra*) (Zhang et al., 2016) and nearly 30 different QTLs in various plant populations harboring resistance to multiple *P. brassicae* strains (Manzanares-Dauleux et al., 2000; Werner et al., 2008; Li et al., 2016; Laperche et al., 2017; Aigu et al., 2018; Hejna et al., 2019; Wagner et al., 2019). A series of studies by our group using a DH population derived from the varieties ‘Darmor-*bzh*’ and ‘Yudal’ allowed the identification of a major QTL controlling resistance to *P. brassicae* isolate Pb137-522, while two QTLs were found to govern resistance to isolate K92-16 (Manzanares-Dauleux et al., 2000). In addition, when this DH population was tested against the pathogen isolate “eH,” two major and one minor QTLs were found to control resting spore production (Aigu et al., 2018), one moderate and three minor QTLs controlled the pathogen–plant genomic DNA ratio (Wagner et al., 2019), and nine QTLs regulated clubroot severity, measured as a DI (Laperche et al., 2017; Aigu et al., 2018; Wagner et al., 2019). Moreover, other groups have identified different QTLs controlling clubroot resistance in one DH population challenged with seven different isolates of *P. brassicae* (Werner et al., 2008), in an associative transcriptome analysis of 245 accessions inoculated with the European Clubroot Differential (ECD) pathotype 17/31/31 (Hejna et al., 2019), and in a genome wide association study of 472 host accessions inoculated with Williams’ pathotype 4 (Li et al., 2016).

Previous studies have included very diverse genetic materials (spring, old winter lines), whose use in breeding programs may be difficult given potential issues such as linkage drag (Zamir, 2001; Yousef and Juvik, 2002; Lecomte et al., 2004). The challenges associated with the introduction of polygenic partial resistance from non-elite materials into elite oilseed rape genetic backgrounds may be one reason for the limited introduction of QTLs into new CR cultivars. Therefore, the identification of loci conferring partial clubroot resistance in recent cultivars with good agronomic performance may be of interest, as breeders could use these more readily.

Sustainable clubroot management requires both short- and long-term approaches. In the short term, the focus should be on minimizing the impact of the pathogen on the host and therefore on crop production; in the longer term, the aim should be on reducing inoculum levels. Resistant cultivars are a convenient and highly effective disease management tool in the short term, but may not prove durable over the long term. The loss of effective pathogen control may reflect the strong selection pressure imposed on the pathogen, particularly since plants with low disease severity do not necessarily produce low amounts of resting spores (Siemens et al., 2002). A recent evaluation of *P. brassicae* resting spore dynamics in response to the cropping of CR *B. napus* cultivars indicated increases in soil inoculum loads (Ernst et al., 2019).

Plant disease epidemics are highly influenced by pathogen virulence (measured as disease severity) and fitness (measured as the reproductive rate of the pathogen), both of which are generally assumed to be positively correlated (Sacristán and García-Arenal, 2008); clubroot is no exception. However, studies with respect to the relationship between pathogen virulence and reproductive rate have produced contradictory results in some pathosystems (Fox and Williams, 1984; Kover and Schaal, 2002; Montarry et al., 2006). Indeed, there are reports where pathogen inoculum production and virulence are not correlated, or even negatively correlated (Robert et al., 2002; Sacristán et al., 2005; Montarry et al., 2010; Aigu et al., 2018).

Given the assumption of a high correlation between disease severity and pathogen fitness, most genetic studies of clubroot resistance have focused only on disease severity (Manzanares-Dauleux et al., 2000; Werner et al., 2008; Laperche et al., 2017). However, while there is a relationship between *P. brassicae* resting spore production and root gall size (disease severity), these variables are not necessarily correlated, and resting spore production can be affected by host resistance and environmental factors (Murakami et al., 2004; Gravot et al., 2016; Aigu et al., 2018). Therefore, both traits should be considered for genetic analyses and in breeding programs. The new CR ideotypes should combine low resting spore production and low DI. Selection for both traits will facilitate improved disease management over the short and long term. In the short term, such an approach will minimize the direct impact of the pathogen on disease severity and hence on crop production, while in the long term, it will reduce the number of resting spores released into the soil, thereby limiting inoculum build-up and the potential for future epidemics.

This research had two objectives. First, it aimed to identify the QTLs involved in the control of resting spore production per plant (RSP) and clubroot symptoms (DI) in a segregating DH population from the cross of ‘Aviso’ × ‘Montego,’ two modern winter oilseed rape cultivars partially resistant to clubroot with good agronomic characteristics. Second, it aimed to identify some recombinant lines of potential interest for breeding efforts, i.e., carrying favorable alleles at multiple QTLs to decrease DI and limit resting spore production.

## Materials and Methods

### Pathogen Material and Inoculum Preparation

The *P. brassicae* selection isolate “eH” (Fähling et al., 2003), classified as pathotype P_1_ on the system of Somé et al. (1996), was used for all the experiments. To prepare inoculum, resting spores were extracted from frozen root galls of the universally susceptible Chinese cabbage (*B. rapa* subs. *pekinensis*) cv. Granaat (ECD 05; Buczacki et al., 1975) inoculated with the isolate. Briefly, the galls were thawed at room temperature and then homogenized at maximum speed in a home blender. The resulting homogenate was filtered sequentially, first through cheesecloth and then through 56 and 100-μm diameter pore stainless steel sieves (Retsch, Haan, Germany). The resting spore concentration of the filtered suspension was estimated by counting in a Malassez cell and adjusted to a final concentration of 1 × 10^7^ resting spores ml^–1^ with sterile distilled water.

### Greenhouse Experiments and Disease Assessment

A 2-year experiment was conducted under greenhouse conditions in 2015 and 2016. The experimental design consisted of completely randomized blocks nested within the 2 years; in total, 114 genotypes (treatments) with four replicates (blocks) were established, with the experimental unit comprising six plants per genotype. Seeds of each genotype were sown in 4-cm-diameter pots (one seed per pot) filled with “Falienor 922016F3” potting mix (Falienor, Vivy, France), which consists of 65% Irish peat, 20% black peat, 15% perlite, and 2% clay (pH = 6.2). The greenhouse was maintained at temperatures between 19 and 22°C under a 16/8 day/night cycle. Plants were fertilized with “Liquoplant FD 134 hiver” nutrient solution (Plantin, Courthéson, France) once or twice a week by sub-irrigation. Inoculations were conducted 7 days after sowing, by applying 1 ml of the *P. brassicae* resting spore suspension to the base of each seedling.

Disease assessment was conducted 54 dai on a 0–3 scale following Manzanares-Dauleux et al. (2000), where 0 = no visible galling, 1 = very light galling usually confined to lateral roots, 2 = moderate galling on lateral roots and the taproot, 2 + = severe galling on all roots but some roots remain healthy, and 3 = one large gall with no remaining healthy roots. The individual severity ratings were then used to calculate a DI using Eq. 1:

$$D\,I=\frac{\overset{[\left(0\times n_{0}\right)+\left(25\times n_{1}\right)+\left(50\times n_{2}\right)+\left(75\times n_{2+}\right)}{+\left(100\times n_{3}\right)]\times 100}}{N}$$

Where *n*_*0*_, *n*_*1*_,*n*_2_,*n*_2 +_, and *n*_*3*_ represent the number of plants in each severity class and *N* is the total number of plants evaluated. To confirm the pathotype designation of the isolate “eH” as P_1_, the isolate was inoculated on the differential hosts of Somé et al. (1996) [*B. napus* cv. Nevin (ECD 06), *B. napus* cv. Wilhelmsburger (ECD 10), and *B. napus* cv. Brutor]. The Chinese cabbage ECD 05 was also included as a susceptible check in all of the experiments.

After disease assessment, all roots in the experimental unit were pooled and stored at −20°C until processing. Resting spores in the pooled root samples were quantified by flow cytometry following Aigu et al. (2018). Briefly, the roots were thawed at room temperature and weighted. Each sample was homogenized in 100 ml of distilled water in a home blender, and the resulting suspension was filtered as described for the inoculum preparation. The resting spore suspensions were diluted in a 1:20 ratio with distilled water. Resting spores were quantified in a CyFlow flow cytometer (Sysmex partec, Görlitz, Germany) equipped with a 20-mW blue laser (488 nm) and a Forward-Scatter (FCS) detector to determine particle size (Aigu et al., 2018).

A standard curve was generated from a suspension of resting spores, produced as described above for inoculum preparation, with the spore concentration estimated by counting in a Malassez cell. This solution was then diluted as needed to generate nine spore suspensions with concentrations ranging from 1 × 10^4^ to 6 × 10^5^ resting spores⋅ml^–1^. Particle counting was completed for each of the dilution points with the flow cytometer and an injection speed of 20. The measured resting spore concentrations were adjusted with a regression Eq. (2) obtained from the standard curve. The obtained regression curve had an *R*^2^ of 95%. Spore levels were expressed as RSP by dividing the total number of resting spores in the suspension by the number of plants in the pooled sample.

(1) $$R\,S=2\times 10^{-6}\,x^{2}+2.2283\,x+41716$$

Where *RS* is the adjusted resting spore count in the suspension and *x* is the particle count obtained with the flow cytometer.

### Plant Material and Genetic Map

A population of 114 DH lines obtained from the cross of the winter oilseed rape cultivars ‘Aviso’ × ‘Montego’ was chosen for linkage analysis because previous experiments indicated that both parents are partially resistant to clubroot, and therefore the presence of new QTLs controlling the disease was suspected. The parental lines were used as controls in all experiments. The genetic map used for the analysis was described by Delourme et al. (2013). Additional genotyping was conducted with the 60K infinitum array (Clarke et al., 2016), thus leading to an updated map. Given the population size, recombination was not always possible, resulting in a high number of markers at the same genetic position. Since this is not desirable for QTL mapping, we only kept a single marker or unique loci to represent each cluster. The genetic map covers 1947 cM (892 cM for the A genome and 1055 cM for the C genome) at a density of 1.18 markers each cM; it comprises 2301 SNPs representing 831 unique loci. The linkage groups (LGs) with the highest proportion of loci distortion were A02, A03, A09, C01, and C09.

### Statistical Analyses

Statistical analyses were performed with R (R Core Team, 2019). Spearman’s rank correlation coefficient was estimated to evaluate the correlation between RSP and DI. The mixed linear model presented in Eq. 3 was estimated to analyze RSP and DI. The *nlme* (Pinheiro et al., 2019) and *lsmeans* (Lenth, 2016) packages were used to test both random and fixed effects:

(2) $$Y_{i\,j\,k}=\mu +G_{i}+Y_{j}+B_{k\,\left(j\right)}+G\,Y_{i\,j}+\varepsilon_{i\,j\,k}$$

Where *Y*_*ijk*_ is either RSP or DI in each genotype (*i*^th^) observed in the *j*^*th*^ year and in the *k*^*th*^block; μ is the population mean; *G*_*i*_ is the effect of the *i*^*th*^ genotype; *Y*_*j*_ is the effect of the*j*^*th*^ year; *B*_*k*(*j*)_ is the effect of the *k*^*th*^ block nested with the *j*^*th*^ year; *GY*_*ik*_ is the effect of the interaction between the *i*^th^ genotype and the *j*^*th*^ year; and ε_*ijk*_ are the residuals. *B*_*k(j)*_ was considered as random.

Broad sense heritability as defined by Holland et al. (2003) (Eq. 4) was estimated using the model in Eq. 3.

(3) $$H^{2}=\frac{\sigma^{2}\,G}{\sigma^{2}\,G+\frac{\sigma^{2}\,G\,y}{y}+\frac{\sigma^{2}\,e}{y*b}}$$

Where *H*^2^ is the broad sense heritability, σ^2^*G* is the variance of the genotype effect, σ^2^*Gy* is the variance of the genotype × year effect, *y* is the number of years, *b* is the number of blocks, and σ^2^*e* is the residual variance.

Adjusted means were calculated using the *lsmeans* package (Lenth, 2016) for each genotype across years and replications and used as phenotypic values for QTL analyses.

The reactions of the progeny were grouped based on genotypes which possessed the identified QTLs for DI and RSP, and the phenotypic responses were compared using Duncan’s new multiple range test at *P* ≤ 0.05.

### Linkage Analysis

Linkage analysis was conducted with the R/qtl package (Broman et al., 2003). At first, a simple interval mapping (SIM) was performed to get a rough estimate of the QTLs controlling each trait (RSP and DI). The LOD threshold for these analyses was 3.17, estimated by a 1000 permutation test (α = 0.05). Afterward, multiple QTL mapping was conducted. Manual selection of the QTL model was completed with the functions *addqtl*, *addintqtl*, and *fitqtl*, allowing for QTL-pairwise interaction using the multiple imputation regression method. The QTLs were added one by one and at each step, and two models were compared: one with the already validated QTLs, and the same model including the tested QTL and the corresponding QTL by QTL interactions. Only QTLs whose effect was significant (α = 0.05) according to the ANOVA table were retained in the model. LOD values and *R*^2^ values for each QTL were also obtained by the *fitqtl* function. The confidence intervals of the QTLs were estimated with a LOD drop of one unit.

For each QTL, the genes present in the confidence interval were gathered using the reference genome v4 of ‘Darmor-*bzh*’ (Chalhoub et al., 2014) by using the physical anchorage of the confidence interval flanking SNP markers.

## Results

### Characterization of the Phenotypic Response

**FIGURE 1:**
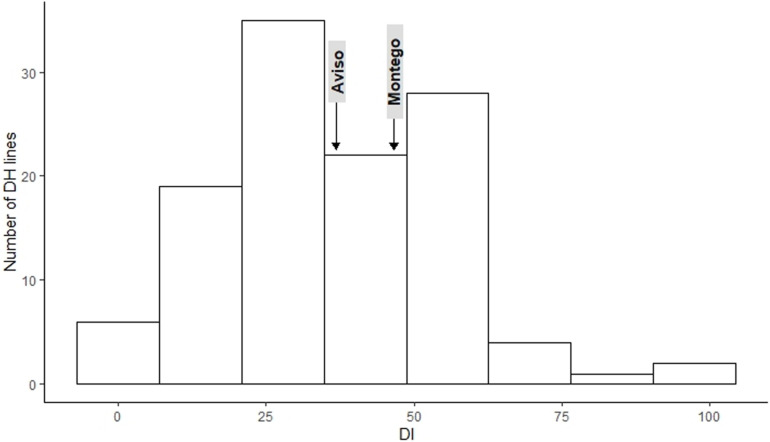
Distribution of the clubroot disease index (DI) for the doubled haploid progeny from a cross of the oilseed rape cultivars ‘Aviso’ × ‘Montego’ following inoculation with *Plasmodiophora brassicae* isolate “eH.” The parents ‘Aviso’ and ‘Montego’ are highlighted.

**FIGURE 2:**
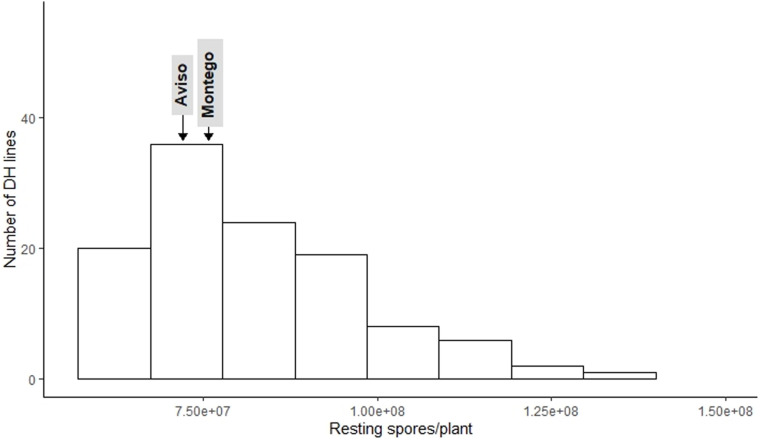
Distribution of number of *Plasmodiophora brassicae* resting spores produced per plant for the doubled haploid progeny from a cross of the oilseed rape cultivars ‘Aviso’ × ‘Montego’ following inoculation with the pathogen isolate “eH.” The parents ‘Aviso’ and ‘Montego’ are highlighted.

**FIGURE 3:**
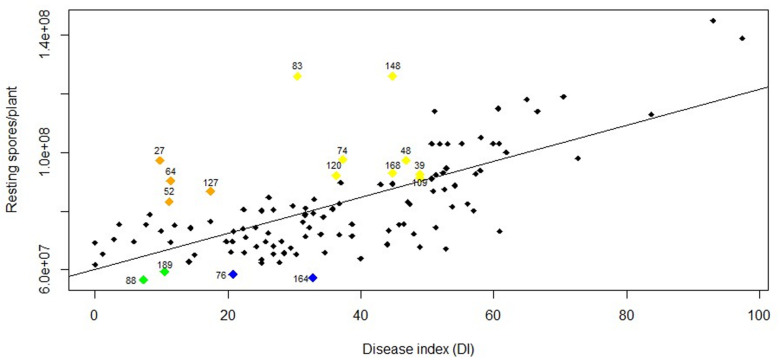
Relationship between disease index (DI) and number of resting spores per plant (RSP) for the doubled-haploid progeny from a cross of the oilseed rape cultivars ‘Aviso’ × ‘Montego.’ The Spearman’s rank correlation coefficient was 0.65 (*p-*value < 0.001). Numbers over the highlighted points indicate the code of each line with atypical behavior. Green points represent lines with a DI < 20 and an RSP < 5.9 × 10^7^ resting spores plant^–1^; blue points represent lines with a DI between 20 and 40 and an RSP < 5.9 × 10^7^ resting spores plant^–1^; orange points represent lines with DI < 20 and an RSP > 8 × 10^7^ resting spores plant^–1^; yellow points represent lines with DI between 30 and 50 and an RSP > 9 × 10^7^ resting spores plant^–1^.

The frequency distribution for DI (Figure 1) and RSP (Figure 2) indicated a continuous distribution, suggesting polygenic control of both variables. In both cases, the parents ‘Aviso’ and ‘Montego’ showed intermediate clubroot responses. Mean DI was 36.8 for ‘Aviso’ and 46.5 for ‘Montego’; the RSP was 7.2 × 10^7^ resting spores plant^–1^ for ‘Aviso’ and 7.6 × 10^7^ resting spores plant^–1^ for ‘Montego.’ RSP was significantly correlated with DI (*p* value < 0.001) (Figure 3), resulting in a Spearman’s coefficient of 0.65. The heritability for both variables was high (85.2% for DI and 84.4% for RSP).

Among the 114 recombinant progeny lines tested, 18% had a DI < 20; 53% had intermediate levels of disease (DI between 20 and 50), while the remaining 29% had a DI between 50.7 and 98. RSP ranged from 5.6 × 10^7^ to 1.4 × 10^8^, with about 54.2% of the lines exhibiting a higher RSP than both parents. Only 30% of the lines produced fewer resting spores than the parental lines (Figure 2).

Lines 88 and 189 developed mild symptoms of clubroot (DI of 7.3 and 10.5, respectively) but had significant resting spore production (RSP of 5.6 × 10^7^ and 5.9 × 10^7^ resting spores plant^–1^, respectively) (Figure 3). Similarly, although lines 27, 64, 52, and 127 had a DI < 20, the RSP was fairly high, ranging from 8 × 10^7^ in lines 52 and 127 to 9 × 10^7^ resting spores plant^–1^ in lines 27 and 64. Some lines with intermediate DI also showed high resting spore production, including line 83 (DI 30.5 and 1.2 × 10^9^ resting spores plant^–1^), line 148 (DI 44.8 and 1.2 × 10^9^ resting spores plant^–1^), line 120 (DI 36.3 and 9.2 × 10^8^ resting spores plant^–1^), line 74 (DI 37.3 and 9.7 × 10^8^ resting spores plant^–1^), line 168 (DI 44.8 and 9.3 × 10^8^ resting spores plant^–1^), line 48 (DI 46.8 and 9.7 × 10^8^ resting spores plant^–1^), and lines 109 and 39 (DI 48.9 and 9.2 × 10^8^ resting spores plant^–1^). In contrast, some lines with an intermediate DI had a lower RSP, including lines 76 (DI 20.8 and 5.8 × 10^7^ resting spores plant^–1^) and 164 (DI 32.8 and 5.7 × 10^7^ resting spores plant^–1^) (Figure 3).

### QTLs Controlling DI and Number of Resting Spores per Plant

**FIGURE 4:**
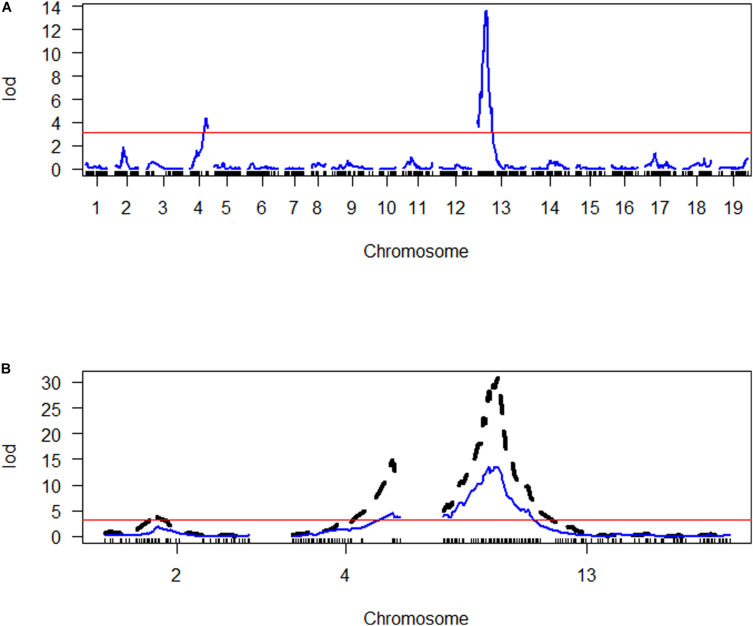
Genome scan of LOD scores for disease index (DI). **(A)** The LOD scores estimated by simple interval mapping (SIM). **(B)** The LOD scores for the chromosomes where QTLs were identified (A02 = chromosome 2, A04 = chromosome 4, C03 = chromosome 13); the LOD scores estimated by multiple QTL mapping are indicated in black, while the LOD scores estimated by SIM are indicated in blue. The red line in both graphs represents the LOD threshold determined by 1000 permutations.

Quantitative trait loci were assigned names consisting of three parts separated by underscores. The first part of each name includes the initials Pb and Bn to indicate *P. brassicae* and *B. napus*, respectively. The second part indicates the trait controlled by the QTL, DI, or RSP, in lowercase letters. The third part indicates the chromosome on which the QTL is located. SIM indicated the presence of two main QTLs controlling DI on chromosomes A04 and C03 (Figure 4), PbBn_di_A04, and PbBn_di_C03. Further analysis allowed the identification of an additional QTL on chromosome A02, PbBn_di_A02, and the final fitted model accounted for 78.4% of the total variation. Most of the variance in DI was controlled by PbBn_di_C03 (51.0%) followed by PbBn_di_A04 (18.1%). A minor effect was detected for PbBn_di_A02, which explained 4.5% of the total variation. Since the population under investigation was a DH, only homozygous lines were tested, and thus only additive effects were detectable, leading to a strong relationship between the percentage of the variance accounted for by the QTLs and their additive effect. It was observed that the QTL with the strongest effect (PbBn_di_C03) also had the highest additive effect (Table 1). No epistasis was found among the QTLs.

**TABLE 1:** QTLs controlling clubroot disease index (DI) or number of *Plasmodiophora brassicae* resting spores per plant (RSP), and their position in the physical map, identified by multiple QTL mapping in a doubled haploid population obtained from a cross of the oilseed rape cultivars ‘Aviso’ × ‘Montego.’

QTL name	Trait	Chromo some	Position (cM)	CI (cM)	Markers CI	LOD	CI (cM)	R^2^ (%)	Favorable allele source	Additive effect	Position in physical map (Mb)	Number of genes	Unannotated or proteins with unknown function	Genes related with plant resistance
PbBn_di_A02	DI	A02	28.9	28.0–34.0	BS008863–BS009106	4.7	28.0–34.0	4.5	Montego	−6.93	4.84–5.50	101	23	5
PbBn_di_A04		A04	57.6	57.6–59.4	BS006202–BS006447	15.2	57.6–59.4	18.1	Montego	−7.737	1.34	.	.	.
PbBn_di_C03		C03	30.8	28.1–32.6	BS007532–Bn-C3-p5080881	30.3	28.1–32.6	51	Aviso	13.193	4.09–4.88	147	29	6
PbBn_rsp_C03	RSP	C03	24.5	19.1–29.0	BS012716–Bn-C3-p4469843	5.1	19.1–29.0	18.3	Aviso	8.5 × 10^5^	2.89–4.50	346	72	10

*CI, confidence interval.*

**FIGURE 5:**
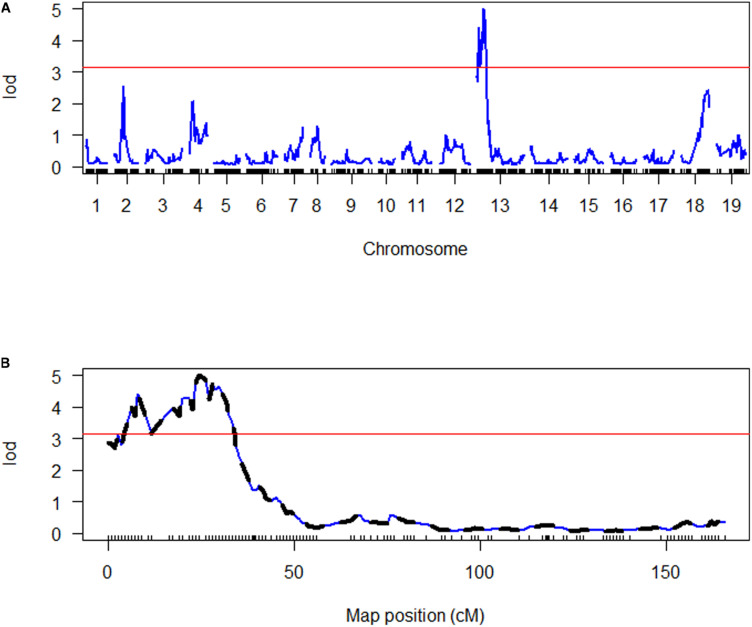
Genome scan of LOD scores for the number of resting spores per plant. **(A)** The LOD scores estimated by simple interval mapping (SIM). **(B)** The LOD scores for the chromosome C03 where one QTL was identified (C03 = chromosome 13); the LOD scores estimated by multiple QTL mapping are indicated in black, while the LOD scores estimated by SIM are indicated in blue. The red line represents the LOD threshold determined by 1000 permutations.

**FIGURE 6:**
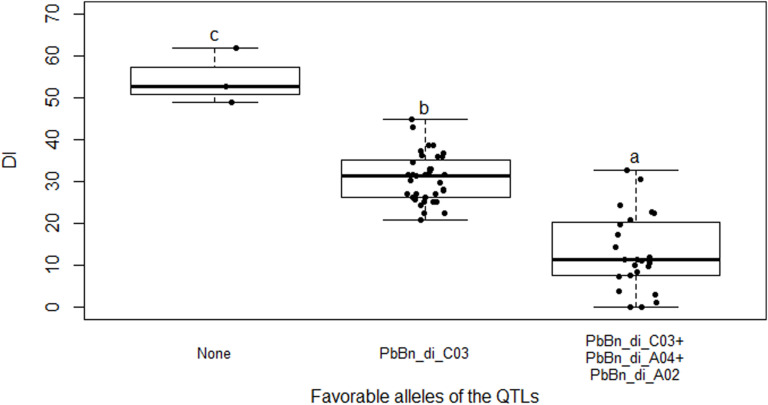
Boxplot showing clubroot disease index (DI) in the doubled-haploid progeny from a cross of the oilseed rape cultivars ‘Aviso’ × ‘Montego.’ The reactions of the progeny are grouped based on genotypes which possess none of the favorable QTL alleles (“None”); the favorable allele of the QTL PbBn_di_C03 (“PbBn_di_C03”); and the favorable alleles of the QTLs PbBn_di_C03, PbBn_di_A04, and PbBn_di_A02 (“PbBn_di_C03 + PbBn_di_A04 + PbBn_di_A02”). Boxes with the same letter within the graph do not differ according to Duncan’s new multiple range test at *p* > 0.05.

Only one QTL controlling RSP, PbBn_rsp_C03 on the chromosome C03, was detected either by SIM or by fitting a multiple QTL model (Figure 5). The fitted model accounted for 18.3% of the total variation for that variable. PbBn_di_C03 and PbBn_rsp_C03 overlapped. On PbBn_di_C03 and PbBn_rsp_C03, the ‘Aviso’ allele contributed to phenotypes with lower DI and RSP, respectively, while on PbBn_di_A04 and PbBn_di_A02, the ‘Montego’ alleles were more favorable for reducing DI (Table 1). The lines that did not have any favorable allele for disease reduction presented the highest DI; an intermediate DI was observed only when the favorable allele of PbBn_di_C03 was present, and the highest reductions in DI occurred whenever the PbBn_di_C03, PbBn_di_A04, and PbBn_di_A02 favorable alleles were present together (Figure 6).

**FIGURE 7:**
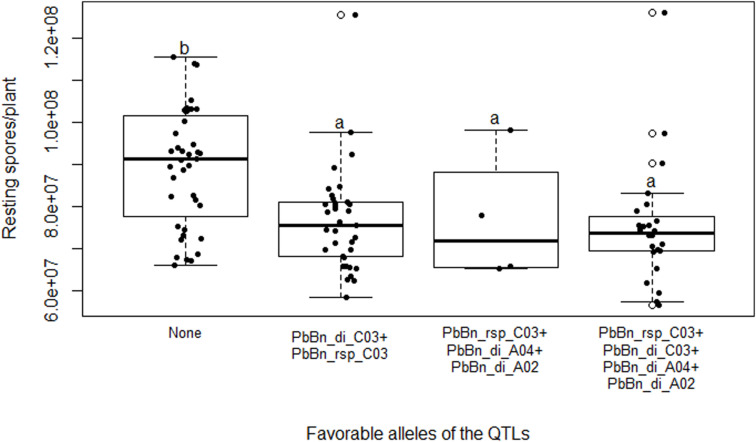
Boxplot showing *Plasmodiophora brassicae* resting spores per plant in the doubled-haploid progeny from a cross of the oilseed rape cultivars ‘Aviso’ × ‘Montego.’ The progeny are grouped based on genotypes which possess the favorable alleles of the QTLs PbBn_rsp_C03, PbBn_di_A04, and PbBn_di_A02 (“PbBn_rsp_C03 + PbBn_di_A04 + PbBn_di_A02”); the favorable alleles of the QTLs PbBn_rsp_C03 and PbBn_di_C03 (“PbBn_rsp_C03 + PbBn_di_C03”); the favorable alleles of the QTLs PbBn_rsp_C03 + PbBn_di_C03 + PbBn_di_A04 + PbBn_di_A02 (“PbBn_rsp_C03 + PbBn_di_C03 + PbBn_di_A04 + PbBn_di_A02”); or none of the favorable alleles (“None”). Boxes with the same letter within the graph do not differ according to Duncan’s new multiple range test at *p* > 0.05.

It was confirmed that RSP was not controlled by the QTLs PbBn_di_A02 or PbBn_di_A04, since the presence of their favorable alleles did not cause a difference in this value (Figure 7).

### Physical Anchorage of the Confidence Intervals on the *B. napus* Genome

The markers names, positions, and start and end-points for all QTLs are indicated in Table 1.

Only three of the four identified QTLs could be mapped on the *B. napus* genome: PbBn_di_A02, PbBn_di_C03, and PbBn_rsp_C03. The QTL PbBn_di_A04 could not be mapped because the region where it was positioned corresponded to a chimeric region on the reference genome (v4). PbBn_di_A02 covered 661.48 kb in a region encoding 101 genes, while PbBn_di_C03 covered 788.420 kb in total, encoding 147 genes. Four genes related to plant resistance and defense were found in PbBn_di_A02 QTL. Two were annotated as hypersensitive-induced response protein (HIR), two as a cyclic nucleotide gated channel, and the fourth as a Mlo-like protein 6. In PbBn_di_C03, six genes related to disease resistance or defense were identified, including five TIR-NBS-LRR class disease resistance proteins and one LRR protein kinase-like protein.

The only QTL related to RSP, PbBn_rsp_C03, covered 1614.43 kb. In this QTL, 346 genes were encountered, of which 10 were related to disease resistance or defense, including five TIR-NBS-LRR class disease resistance proteins, three leucine-rich repeat (LRR) protein kinase-like proteins, one defensin-like protein 203, and one WRKY transcription factor 18. PbBn_rsp_C03 QTL overlapped with PbBn_di_C03 in the region from 28.10 to 29.01 cM (corresponding to a 0.39 Mb physical region). In the common region between PbBn_di_C03 and PbBn_rsp_C03 (4.09–4.50 Mb), three TIR-NBS-LRR class disease resistance proteins were found (Table 2).

**TABLE 2:** Annotation of the genes related to resistance and defense for the QTLs controlling clubroot disease index (DI) or number of *Plasmodiophora brassicae* resting spores per plant (RSP), in a doubled haploid population obtained from a cross of the oilseed rape cultivars ‘Aviso’ × ‘Montego.’

QTL name	Trait	Chromosome	Position in physical map (Mb)	Gene name	Position (bp)	Gene annotation	Homoeologous gene name	*Arabidopsis thaliana* orthologs	*Brassica rapa* ortologs	*Brassica oleracea* ortologs
PbBn_di_A02	DI	A02	4.84–5.50	BnaA02g09860D	4945996–4948245	Hypersensitive-induced response protein (HIR) 2	BnaC02g13810D	AT5G54100	Bra022696	Bo2g041840
				BnaA02g09870D	4950703–4954201	Hypersensitive-induced response protein (HIR) 2	BnaC02g13820D	AT5G54095	Bra022695	Bo2g042150
				BnaA02g09790D	4902667–4907022	Cyclic nucleotide gated channel	BnaC02g44680D	AT5G54250	Bra022702	Bo2g042020
				BnaA02g10440D	5360323–5362868	Cyclic nucleotide gated channel	BnaC02g14560D	AT5G53130	Bra022632	.
				BnaA02g10080D	5102140–5105226	Mlo-like protein 6	BnaC02g14090D	AT5G53760	Bra022673	Bo2g044430
PbBn_di_C03		C03	4.50–4.88	BnaC03g09410D	4510238–4510891	TIR-NBS-LRR class disease resistance proteins	.	.	Bra006487	Bo3g013450
				BnaC03g09420D	4511004–4512486	TIR-NBS-LRR class disease resistance proteins	.	.	.	.
				BnaC03g10100D	4867978–4869203	Leucine-rich repeat protein kinase-like protein	.	AT5G20480	Bra006560	Bo3g015190
PbBn_rsp_C03	RSP	C03	2.89–4.09	BnaC03g08650D	4082610–4087660	TIR-NBS-LRR class disease resistance proteins	.	AT5G17880	.	Bo3g012820
				BnaC03g08660D	4089063–4094327	TIR-NBS-LRR class disease resistance proteins	.	AT5G17890	.	Bo3g012830
				BnaC03g06310D	3057621–3060722	Leucine-rich repeat protein kinase-like proteins	BnaA03g04770D	AT5G14210	Bra006243	Bo3g009670
				BnaC03g07510D	3539026–3542690	Leucine-rich repeat protein kinase-like proteins	BnaA03g05790D	AT5G16000	Bra006335	Bo3g010820
				BnaC03g07920D	3723953–3729620	Leucine-rich repeat protein kinase-like proteins	.	.	Bra006366	Bo3g012140
				BnaC03g08510D	4041438–4041833	Defensin-like protein 203	.	.	.	Bo3g012680
				BnaC03g06770D	3236365–3238839	WRKY transcription factor 18	BnaA03g05230D	AT5G15130	Bra006283	Bo3g010100
Common region between PbBn_di_C03 and PbBn_rsp_C03		C03	4.09–4.50	BnaC03g08900D	4222525–4224460	TIR-NBS-LRR class disease resistance proteins	.	.	.	.
				BnaC03g08920D	4227171–4232582	TIR-NBS-LRR class disease resistance proteins	BnaAnng27760D	AT5G18370	.	.
				BnaC03g09010D	4264274–4265687	TIR-NBS-LRR class disease resistance proteins	BnaA03g07110D	.	Bra006458	Bo3g013130

## Discussion

Research on clubroot resistance has focused mainly on disease severity expressed as a DI. Resting spore production in host genotypes has not been examined to the same extent, although this is an important measurement of pathogen fitness, affecting inoculum build up in infested fields. In the ‘Aviso’ × ‘Montego’ population, the highest observed RSP (1.4 × 10^8^ resting spores plant^–1^) was 2.5 times greater than the lowest (5.6 × 10^7^ resting spores plant^–1^). Such variation may be important from an epidemiological perspective, since doubling the number of resting spores released into the soil could result in faster and more significant inoculum increases for future crops.

Our results indicate that RSP is not as strongly correlated with DI (Spearman’s coefficient of 0.65) as might be expected based on the trade-off hypothesis, where within-host multiplication, within-host transmission, and virulence of pathogens are positively correlated traits (Frank, 1996); and thus, higher disease levels would result in higher within host reproduction rates (Sacristán and García-Arenal, 2008). Nonetheless, these results are consistent with previous reports on the clubroot pathosystem. In a study with *Arabidopsis thaliana*, Siemens et al. (2002) observed that correlation between DI and spore number per root weight was between 0.7 and 0.9, and that one of the main factors determining those correlation values was host resistance. Similarly, Murakami et al. (2004) concluded that resting spore production is host-specific and, therefore, clubroot severity cannot account for resting production on its own. This was especially true for intermediate disease severities; for example, Chinese cabbage and broccoli plants with intermediate levels of clubroot produced approximately 1 × 10^9^ resting spores plant^–1^, while cabbage plants with the same amount of disease produced only 1 × 10^8^ resting spores plant^–1^ (Murakami et al., 2004). More recently, Aigu et al. (2018) reported that *P. brassicae* was able to produce high numbers of resting spores in some *B. napus* genotypes with mild symptoms of clubroot, and highlighted the partially resistant genotype ‘Darmor*-bzh*’ (2.7 × 10^8^ resting spores plant^–1^ and DI = 30).

Linkage analysis showed that the genetic control of RSP and DI are related, since the QTLs PbBn_di_C03 and PbBn_rsp_C03 co-localized, suggesting that the C03 locus controls both traits. The importance of this genomic region is highlighted by the fact that PbBn_di_C03 explains the highest proportion of the variation for DI. The other loci, PbBn_di_A04 and PbBn_di_A02, only control DI and not RSP. We have reported this type of genetic architecture previously, where all QTLs controlling RSP also control DI, but not all DI QTLs control RSP (Laperche et al., 2017; Aigu et al., 2018). Co-localization of two QTLs with an intermediate effect on DI (QTL controlling < 20% of the variation) and a high effect on RSP (QTL controlling > 50% of the variation) has also been reported (Laperche et al., 2017; Aigu et al., 2018). Collectively, these studies suggest that co-localization of DI and RSP QTLs does not depend on the QTL effect (percentage of the variance of the trait that the QTL explains).

In a recent study of the genomic regions controlling DI and RSP in response to *P. brassicae* isolate “eH” in another *B. napus* DH population (‘Darmor-*bzh*’ × ‘Yudal’), five QTLs controlling DI were located on chromosomes A05, A07, C02, C03, and C09 (Laperche et al., 2017). Two of these QTLs, on chromosomes C02 and C09, were also found to control RSP (Aigu et al., 2018). In the current study, we also identified QTLs on chromosome C03, but ours were in the regions 4.09–4.88 Mb (PbBn_di_C03) and 2.89–4.5 Mb (PbBn_rsp_C03), while the one found by Laperche et al. (2017) occurred in the region 4.6–5.0 Mb. This suggests that the same genomic region may be controlling clubroot resistance in both populations; however, further analysis is required for confirmation. The percentage of the variation explained by PbBn_di_C03 in our study and the QTL on chromosome C03 reported by Laperche et al. (2017) in the ‘Darmor-*bzh*’ × ‘Yudal’ population was very different. The QTLs we detected on C03 controlled 51.4% of DI and 18.3% of the variation in RSP, while Laperche et al. (2017) found that the QTL on chromosome C03 controlled 7.75% of DI. No QTL controlling RSP was found on chromosome C03 in the ‘Darmor-*bzh*’ × ‘Yudal’ population (Aigu et al., 2018). Similarly, Werner et al. (2008) found QTLs in the LGs N13 and N02 (chromosomes C03, and A02, respectively) in a DH population of *B. napus* obtained from a cross of the DH line 263/11 and the oilseed rape cultivar ‘Express’ challenged with the *P. brassicae* isolates “*01:60*,” “*01:07*,” and “*k*.” QTLs on chromosome C03 were identified when plants were inoculated with isolates “*01:60*” and “*01:07*” and explained 28.6 and 11.7% of the variance in DI, respectively. A QTL on chromosome A02 was also found after inoculation with the isolate “*k*,” explaining 17.6% of the variance in DI. In addition, QTLs at syntenic positions have been identified in *B. oleracea* on chromosome C03 (Nagaoka et al., 2010; Lee et al., 2016; Li et al., 2016) and in *B. rapa* on chromosome A02 (Yu et al., 2017), suggesting that the C03 genomic region merits further investigation. Indeed, the QTLs on chromosome C03 seem to be involved in the genetic control of response to different isolates, as has been observed in multiple genetic backgrounds harboring different effects (minor to major) depending on both the plant genotype and the *P. brassicae* isolate.

Most genetic analyses of the resistance harbored by oilseed rape against clubroot disease have been performed with very diverse genetic material (spring, old winter lines), and few resistance sources appear to be lines with good agronomic value. In the current study, the parents of the DH population were the cultivars ‘Aviso’ and ‘Montego,’ with the former released by Danisco seeds (Holeby, Denmark) in 2000 and the latter released by Limagrain (Saint-Beauzire, France) in 2002. ‘Aviso’ is a variety with good agronomic performance that shows resistance to other diseases such as blackleg (*Leptosphaeria maculans*) (Stonard et al., 2007; Jestin et al., 2015). These cultivars may represent suitable donors of clubroot resistance in oilseed rape breeding programs, considering that with their good agronomic value, less intensive backcrossing with elite lines would be required, facilitating the transfer of polygenic traits into new cultivars.

The potential utility of the ‘Aviso’ × ‘Montego’ population in breeding programs is underscored by the fact that the resting spore production was lower relative to values reported in previous studies. The ‘Aviso’ × ‘Montego’ DH population produced between 5.6 × 10^7^ and 1.45 × 10^8^ resting spores plant^–1^. In contrast, in the ‘Darmor-*bzh*’ × ‘Yudal’ DH population, the RSP values ranged from 1 × 10^8^ and 5 × 10^9^ (Aigu et al., 2018). Hence, the ‘Aviso’ × ‘Montego’ DH population used in this research appears promising for the selection of parental lines with low DI and RSP (lines 189 and 88) for the transfer of the identified QTLs into new cultivars. While it has been reported that the simultaneous transfer of even four unlinked QTLs is possible (Hospital and Charcosset, 1997; Lecomte et al., 2004; Steele et al., 2006), the use of at least three markers for each QTL is recommended to make them useful in marker-assisted selection (Hospital and Charcosset, 1997). The transfer of the four QTLs identified in the current study is therefore possible, although additional refinement and validation of the markers is required for marker-assisted selection.

The search we conducted for the genes underlying the QTLs in the ‘Darmor*-bzh*’ reference genome allowed the identification of a set of genes, which might be involved in the response of the ‘Aviso’ × ‘Montego’ population to *P. brassicae*, on chromosomes A02 and C03. Several of the putative gene products belonged to protein families involved in plant defense and resistance, including HIRs, cyclic nucleotide gated channel, Mlo-like protein 6, TIR-NBS-LRR (TNL) class disease resistance proteins, LRR protein kinase-like, defensin-like protein 203, and WRKY transcription factor 18 (Rushton et al., 1995; Song et al., 1995; Buchanan and Gay, 1996; García-Olmedo et al., 1998; Clough et al., 2000; Eulgem et al., 2000; Dangl and Jones, 2001; Kanzaki et al., 2002; Choi et al., 2010; Zhou et al., 2010; Duan et al., 2013). The presence of a cluster of seven TNL-encoding genes is of special interest. The two clubroot-resistance loci (*CRa* and *Crr1*) that have been molecularly resolved so far (Ueno et al., 2012; Hatakeyama et al., 2013) both encode for TNLs. In addition, the fine mapping of the *Rcr1* locus (Yu et al., 2016) and GWAS have highlighted the recurrent presence of nucleotide-binding LRR (NLR)-gene clusters in clubroot-resistance loci, supporting the importance of this protein family in driving clubroot resistance (Stotz et al., 2018).

The ‘Aviso’ × ‘Montego’ population characterized in this study holds promise for the development of new CR oilseed rape cultivars, since the parental lines were recent varieties with good agronomic traits, and some of the tested lines presented very low DI accompanied by low RSP. While much of the emphasis in clubroot resistance breeding traditionally has been on selection based on low disease severity, resting spore production in plant material should also be considered. While breeding based on DI is critical for producing cultivars that perform well in *P. brassicae*-infested fields, the incorporation of lower RSP as a selection criterion will enable more sustainable clubroot management, by selecting plant genotypes where pathogen multiplication (fitness) is reduced.

## Data Availability Statement

The raw data supporting the conclusions of this article will be made available by the authors, without undue reservation.

## Author Contributions

AB-R conducted some of the laboratory analyses, performed the statistical and linkage analyses, and wrote the manuscript. AL, AG, and MM-D contributed to development of the research concept and design of the study and reviewed multiple versions of the manuscript. AL and MJ designed and directed the execution of the experiments. SG and MJ helped to carry out the clubroot tests. SS provided project guidance and extensively edited the manuscript. All authors contributed to manuscript revision, and read and approved the submitted version

## Conflict of Interest

The authors declare that the research was conducted in the absence of any commercial or financial relationships that could be construed as a potential conflict of interest.

## Abbreviations

CR: clubroot-resistant
dai: days after inoculation
DH: doubled haploid
DI: disease index
RSP: resting spores per plant.
